# Enhancing AI competence in health management: students’ experiences with ChatGPT as a learning Tool

**DOI:** 10.1186/s12909-024-05595-9

**Published:** 2024-05-30

**Authors:** Lior Naamati-Schneider

**Affiliations:** Health Systems Management Department, Hadassah Academic College, Jerusalem, Israel

**Keywords:** AI digital literacy, ChatGPT in education, Healthcare management education

## Abstract

**Background:**

The healthcare industry has had to adapt to significant shifts caused by technological advancements, demographic changes, economic pressures, and political dynamics. These factors are reshaping the complex ecosystem in which healthcare organizations operate and have forced them to modify their operations in response to the rapidly evolving landscape. The increase in automation and the growing importance of digital and virtual environments are the key drivers necessitating this change. In the healthcare sector in particular, processes of change, including the incorporation of artificial intelligent language models like ChatGPT into daily life, necessitate a reevaluation of digital literacy skills.

**Methods:**

This study proposes a novel pedagogical framework that integrates problem-based learning with the use of ChatGPT for undergraduate healthcare management students, while qualitatively exploring the students’ experiences with this technology through a thematic analysis of the reflective journals of 65 students.

**Results:**

Through the data analysis, the researcher identified five main categories: *(1) Use of Literacy Skills; (2) User Experiences with ChatGPT; (3) ChatGPT Information Credibility; (4) Challenges and Barriers when Working with ChatGPT; (5) Mastering ChatGPT-Prompting Competencies*. The findings show that incorporating digital tools, and particularly ChatGPT, in medical education has a positive impact on students’ digital literacy and on AI Literacy skills.

**Conclusions:**

The results underscore the evolving nature of these skills in an AI-integrated educational environment and offer valuable insights into students’ perceptions and experiences. The study contributes to the broader discourse about the need for updated AI literacy skills in medical education from the early stages of education.

## Introduction

 In recent years, the healthcare sector has undergone significant shifts in both local and global contexts. These shifts are primarily attributed to demographic, technological, economic, and political factors. These changes have had a profound impact on the healthcare ecosystem, requiring organizations to adapt their operations and strategies to this evolving landscape [1, 2]. In response, healthcare organizations have had to modify their behavior to adapt to this ever-changing reality [3]. Among the factors that have most significantly affected the healthcare system are technological advancements, automation, and the rise of digital and virtual environments. The impact of these factors gained momentum in December 2019, primarily due to the COVID-19 pandemic. Technological advances, particularly the rise of artificial intelligence (AI) and digital tools, have been central to this transformation, with the COVID-19 pandemic accelerating the need for healthcare systems to adapt and innovate [3–8]. The integration of AI in healthcare, including the deployment of chatbots like ChatGPT that utilize the Generative Pre-trained Transformer (GPT)—a type of large language model (LLM)—underscores a shift toward digital and AI literacy in medical education and practice. [9, 10].

The adoption of AI in healthcare, highlighted by the use of systems like ChatGPT, marks a pivotal shift towards greater digital and AI literacy in medical education and practice [9–12]. This reflects the healthcare sector’s broader move towards technological innovation, aiming to enhance patient care and revolutionize healthcare professional training. Incorporating AI, such as ChatGPT, into educational frameworks prepares students for the complexities of modern healthcare, demonstrating AI’s potential to transform both healthcare delivery and professional skill development [11, 12].

In the rapidly evolving landscape of AI, where technological developments are occurring at an accelerated pace, there is a significant need for comprehensive research to navigate this ever-changing landscape. In particular, research into the impact of AI on healthcare is still limited, highlighting the urgent need for more focused studies on the implications for medical education and the effective training of healthcare professionals in the use of AI technologies [13, 14]. The emergence of LLMs, such as GPT, and their applications in educational frameworks, including chatbots like ChatGPT, has increased the urgency of reassessing the skills required, with a particular focus on digital literacy. This reassessment is essential to determine the continued relevance of these skills or whether a fundamental refocusing is required. Such a re-examination is essential to ensure that the healthcare workforce is adequately prepared for the challenges and opportunities presented by the integration of AI into healthcare practice [11]. 

Studies [15–18] have identified a significant gap in understanding how digital literacy skills—such as accessing, analyzing, evaluating, and creating digital content—play a role in effectively leveraging LLMs like GPT and their applications, including chatbots such as ChatGPT, within educational frameworks. Furthermore, the successful integration of ChatGPT into educational settings may potentially lessen the reliance on traditional digital literacy skills, prompting a reevaluation of their ongoing relevance [19, 20]. This gap underscores the need for more research into the critical role that digital literacy skills hold in the efficient use of technologies like ChatGPT for educational aims, as highlighted by recent literature [15, 17, 18]. ChatGPT’s access to accurate medical information could reduce the need for individual data analysis skills [21, 22]. Yet, concerns persist among researchers that its content generation might hinder critical thinking development, including source evaluation and idea generation [23, 24].

This qualitative study introduces a pedagogical framework that synergizes problem-based learning with the application of ChatGPT among undergraduate healthcare management students. It aims to qualitatively examine their interactions with this technology, focusing on the transition from traditional digital literacy towards a more advanced AI literacy. This evolution in educational focus is poised to revolutionize the requisite competencies for navigating the dynamic healthcare sector of today.

The rationale behind focusing on ChatGPT stems from its notable accessibility, user-friendly design, and versatility as a comprehensive tool in healthcare settings. Its capability to simulate human-like dialogues positions it as a prime resource for educational initiatives, thereby enriching the pedagogical domain of healthcare management and clinical practices. The unrestricted access to ChatGPT, along with its wide-ranging utility in executing diverse healthcare operations, underscores its capacity to significantly contribute to and spearhead innovation within healthcare education and practices. The selection of ChatGPT, attributed to its approachability and adaptability, marks a strategic endeavor to investigate the impact of artificial intelligence amidst the shifting paradigms of healthcare requirements. Yet, despite the widespread integration of ChatGPT in healthcare, research into the long-term effects and the necessary adaptation of skills and methods remains lacking. [11, 12].

### Literature review

#### AI tools in medical settings

AI involves creating systems that mimic human cognitive functions such as perception, speech recognition, and decision-making through machine learning. It excels in analyzing data, identifying patterns, and making predictions, offering improvements over traditional data processing. AI’s applications span multiple sectors, including healthcare, at various levels from individual to global [25, 26]. The integration of AI into healthcare enhances diagnostic, treatment, and patient care, offering advanced decision-making and predictions [9, 10, 25, 27].AI technologies enhance clinical decision-making, diagnosis, and treatment by analyzing patient data through machine learning for informed decisions, offering 24/7 support via AI chatbots, and enabling remote monitoring with AI-powered devices like wearable sensors [9, 28].

AI facilitates remote patient monitoring, minimizing in-person healthcare visits [29]. It improves service personalization, with AI assistants managing appointments and reminders, and chatbots streamlining insurance claims, easing provider workloads [9]. AI automates routine administrative tasks, freeing providers to concentrate on patient care. It streamlines operations, cuts bureaucracy, and analyzes data to improve healthcare management and predict service demand, allowing for better resource allocation. AI’s analysis of patient feedback further aids in enhancing service delivery [10]. AI integration can transform patient-caregiver dynamics, enhancing diagnosis, treatment, and self-management of health conditions [30]. While AI integration in healthcare promises significant advancements, it presents challenges, including data management issues and the need for specialized skills.

Sallam [14] highlights ChatGPT’s potential advantages in healthcare, including enhancing clinical workflows, diagnostics, and personalized medicine. However, challenges such as ethical dilemmas, interpretability issues, and content accuracy must be tackled. In healthcare education, although ChatGPT holds promise for customized learning and creating lifelike clinical scenarios, concerns about bias, plagiarism, and content quality persist. Addressing these concerns necessitates preparing healthcare professionals and students through education and training to navigate the complexities of AI. Additionally, extensive research in these domains is essential [6, 9, 14, 31, 32].

#### Teaching with AI and about AI: advancing education in the digital age

To be able to utilize AI tools effectively and integrate them seamlessly into their everyday work, healthcare professionals need early exposure to AI tools in their education to boost their proficiency and confidence, understanding both their potential and limitations [9, 32, 33]. York et al. [32] explored medical professionals’ attitudes towards AI in radiology, revealing a positive outlook on AI’s healthcare benefits but also highlighting a notable gap in AI knowledge. This emphasizes the need for enhanced AI training in medical education.

According to Sallam [14], ChatGPT and other models based on lLLMs have significantly improved healthcare education. They customize responses to student inquiries, curate relevant educational material, and tailor content to individual learning styles. For instance, ChatGPT generates personalized quiz questions, suggests resources to fill knowledge gaps, and adjusts explanations to suit diverse learning preferences. Moreover, it simplifies complex medical concepts, employs analogies and examples for clarity, and offers supplementary materials to enhance comprehension.

Breeding et al. [11] argued that in medical education, ChatGPT should be viewed as a supplementary tool rather than a substitute for traditional sources. While it offers clear and organized information, medical students still perceive evidence-based sources as more comprehensive. Eysenbach [33] engaged in a series of dialogues with ChatGPT to explore its integration into medical education. ChatGPT demonstrated proficiency in various tasks, such as grading essays, providing feedback, creating virtual patient scenarios, enhancing medical textbooks, summarizing research articles, and explaining key findings. Nevertheless, it also demonstrated a tendency to produce erroneous responses and fabricated data, including references. Such inaccuracies have the potential to generate student misconceptions, spread misinformation, and cause a decline in critical thinking skills [33]. Han et al. [34] conducted a comprehensive examination of ChatGPT’s effectiveness as a pedagogical tool in medical education, focusing on the chatbot’s interaction with delineated educational objectives and tasks. Their findings suggest that while ChatGPT is capable of providing elementary data and explanations, it is not impervious to constraints and sometimes provides incorrect or partial information. The study stresses active learning and analytical reasoning in medical education, emphasizing the importance of understanding basic sciences and the need for expert oversight to ensure AI-generated information accuracy [34].

Das et al. [35] evaluated ChatGPT’s efficacy in medical education, focusing on microbiology questions at different difficulty levels. They found that ChatGPT could answer basic and complex microbiology queries with roughly 80% accuracy, indicating its potential as an automated educational tool in medicine. The study underscores the importance of ongoing improvements in training language models to enhance their effectiveness for academic use [35, 36].AI implementation in healthcare must be carefully managed to maximize benefits and minimize risks [11, 12, 35, 36]. With the rapid development of digital technologies and AI tools, particularly in healthcare, students need appropriate resources to use these technologies effectively [37]. Digital literacy is essential in the 21st century, including skills for interacting with digital content [16, 18]. Hence, medical literacy skills should start early in the education of healthcare students.

#### Digital literacy and eHealth literacy skills

Digital literacy skills encompass a collection of essential abilities necessary for using digital technologies effectively in accessing and retrieving information [38]. These skills are often viewed as foundational digital literacies that are critical for full participation in the digital era [39]. The European Commission emphasizes the importance of digital literacy for employability and citizenship. They advocate for policies and programs to enhance digital skills across all segments of society. The EU aims for 70% of adults to have basic digital skills by 2025, focusing on analytical, evaluative, and critical thinking abilities crucial for assessing digital information’s quality and credibility [40]. Individuals need these skills to discern biases and misinformation in various media formats [16, 17, 41] and evaluate the credibility of online sources [42]. Critical thinking is crucial for distinguishing between accurate information and misinformation [43], while data literacy is essential for interpreting data and detecting misleading statistics [44]. These competencies are fundamental for navigating today’s complex digital information landscape.

eHealth literacy, which incorporates the digital skills needed to access and utilize medical information from digital platforms [45], is gaining recognition as an integral component of overall health literacy. Enhanced online medical literacy is vital for healthcare professionals and administrators [46] to adapt to changing demands and improve care management within evolving healthcare paradigms [47]. Additionally, acquisition of digital competencies has been identified as a valuable strategy that healthcare providers and managers can use to manage the psychological effects of heightened workloads and uncertainty, such as the fear, stress, and anxiety emerging from the COVID-19 pandemic [48]. These skills enable individuals to use AI as both an independent tool and a supplementary aid in decision-making. However, addressing challenges like bias and academic integrity is crucial when integrating AI into medical education [32, 33, 49]. Critical thinking skills are essential for analyzing digital information, identifying inconsistencies, and evaluating arguments. In today’s era of misinformation, users must verify the accuracy of online content and distinguish between reliable sources and hoaxes [43]. Data literacy skills are also crucial for interpreting data accurately, detecting misleading statistics, and making informed decisions based on credible sources in the digital age [44].

Research on digital literacy emphasizes the importance of analytical and evaluative skills. Morgan et al. [17] found that higher education students struggle most with evaluating digital content for bias and quality. They excel in social literacy skills like communication. This highlights the need to prioritize adaptability in digital literacy, integrating industry-relevant experiences into education to ensure students can navigate and critically assess digital information for real-world applications.

Indeed, since the introduction of ChatGPT in 2022, it has been beneficial in various educational contexts. Nevertheless, concerns have been raised about potential inaccuracies and misinformation that may affect student learning and critical thinking [20]. Moreover, the potential redundancy of certain digital skills as a result of ChatGPT’s capabilities has also sparked discussions on changing educational objectives [19, 21, 22]. The development of ChatGPT may replace some digital skills as it takes over tasks previously expected of students. Researchers [21, 22] argue that it is constantly improving its ability to access accurate medical information, providing reliable advice and treatment options from reputable sources. This ability may render the need for individuals to be adept at information retrieval and evaluation redundant. In other words, ChatGPT’s growing proficiency in tasks such as translation, text summarization, and sentiment analysis, and its ability to generate content like movies [23] may potentially lead to the underdevelopment of critical thinking skills, including the ability to evaluate source quality and reliability, formulate informed judgments, and generate creative and original ideas [24]. Indeed, the integration of AI into the healthcare sector raises critical questions about the nature and scope of the digital skills required in the future [19, 20].

As AI advances, essential digital competencies may need reassessment to keep pace with technology. This requires forward-thinking digital literacy initiatives, particularly in healthcare education and practice. Proactively addressing the potential impact of AI on human interactions with digital healthcare technologies is critical. This will ensure that healthcare professionals and students are skilled in current digital practices, and prepared for the evolving role of AI in the sector. Despite the swift integration of AI tools in healthcare, and applications like ChatGPT, research on their long-term impacts, effects on users, and the necessary adaptation of skills and methodologies in the ever-evolving learning environment remains insufficient [11, 12, 15, 17, 18, 49–58].

### This study

This study aims to address the intersection of AI adoption in healthcare and its implications for medical education, specifically focusing on the skills required by healthcare professionals. With the rapid incorporation of AI, into healthcare settings, there is an urgent need to reassess the digital literacy skills traditionally emphasized in medical education. This reassessment prompts questions about the ongoing relevance of these skills as AI technologies continue to evolve and expand their role in healthcare [13, 15–20].

#### Research questions

Given the context, this study aims to explore the following qualitative research questions:

1. How does a pedagogical framework integrating problem-based learning with ChatGPT affect healthcare management undergraduates’ digital literacy skills?
2. What are students’ experiences with the combined use of problem-based learning and ChatGPT in their healthcare management education?
3. How do students perceive the shift towards AI-relevant skills as a result of engaging with this integrated pedagogical approach?

## Methodology

### Methodological approach

The present research adopts the case study methodology, which entails in-depth empirical research of a current phenomenon within its real-world context [50]. This approach involves collecting data on human activities within a defined time and space setting, thereby facilitating an understanding of the various processes occurring within the research case. In qualitative research, and particularly in case study research, themes are formulated from the participants’ narratives, thus allowing for the development of arguments or generalizations derived deductively from participants’ statements [51]. By focusing on our research questions and using a methodological framework that emphasizes depth and context, the study aims to shed light on the transformative impact of AI on medical education and the development of the skills required for future healthcare professionals.

The research was conducted and analyzed by the researcher, who has a PhD in Healthcare Management and over 15 years of experience in qualitative analysis. Her expertise ensures a deep understanding of the study’s qualitative data. Throughout the research, she engaged in continuous reflexive practices to evaluate how her subjectivity and context influenced the study. This included reflecting on her assumptions, considering power dynamics with participants, aligning research paradigms and methods, and understanding the research context [59].

### Participants and research population

The study involved 89 third-year undergraduate students enrolled in a Health System Management degree program, specifically participating in a course on Service Quality in the Healthcare System during the 2023 academic year. The researcher, serving as the lecturer for this course, integrated writing reflective journals into the curriculum as part of the learning process. Following the course’s conclusion and after grades were distributed, the researcher asked students, in adherence to ethical guidelines, if they consented to have their reflective journals analyzed for research purposes, as outlined in the data collection section. Only students who completed all components of the intervention plan outlined for the class were considered potential participants in the research population.

From this group, qualitative data was extracted from the reflective journals of 65 students who consented to participate. The demographic breakdown of this participant subset included 80% females, with an average age of 24.26 years (Standard Deviation = 3.80).

### Data collection

Throughout the course, participants were required to keep a reflective journal documenting their learning journey, to be submitted at the end of the semester. The aim of writing the journal was to capture their personal perceptions of their learning experience. They were encouraged to articulate various challenges, obstacles, and positive and negative aspects they encountered [52]. Specifically, they were asked to describe the main challenges they faced and the obstacles they overcame, and to provide an introspective account of their experiences. The practice of writing a personal journal not only served as a tool for reflection but also helped them adopt a comprehensive perspective on their educational process [53].

The credibility of the reflective journal prompts was assured by grounding their development in an extensive literature review and expert consultations within the field of healthcare education. This process ensured that the prompts accurately reflected the constructs of interest, facilitating consistent and meaningful student reflections. Content validity was emphasized to ensure the journal prompts were aligned with the study’s objectives and relevant to students’ experiences in healthcare management education. Refinement of these prompts to effectively meet research objectives was facilitated through expert input. A detailed coding scheme was developed, featuring definitions and categories reflecting the study’s aims and insights from the journals. The coding was applied to a subset of journals by the researcher to ensure credibility.

The data were collected from the reflective journals in accordance with the intervention plan outlined in the Instructional Method section. The study carefully complied with several ethical guidelines for research with human subjects. The nature and purpose of the research were fully explained to the students, with particular emphasis on the use of reflective journals to evaluate the intervention plan. The students gave their informed consent and signed consent forms. To ensure confidentiality, participants were informed that all names would be replaced by pseudonyms and all identifying details would be removed from the final research report. They were also explicitly told that the journal entries would be processed anonymously. The research was approved by the college’s Ethics Committee.

### Instructional method procedure (intervention plan)

**Fig. 1:**
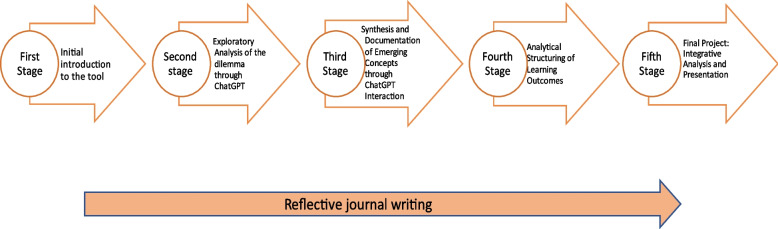
Summary of instructional method

The focus of this study is a required course titled Introducing Quality into the Health System, which had formerly been taught using traditional frontal teaching methods. The study examines the transformation of this course into a course taught using ChatGPT-mediated online guided learning. This innovative learning approach provides learners a comprehensive experience that entails self-directed learning. The approach emphasizes problem-based learning and focuses on identifying ethical dilemmas and analyzing them within organizational contexts. The intervention plan was strategically organized into five primary stages. Each stage comprised a series of carefully constructed steps that were specifically designed to build upon the knowledge and skills acquired in the previous stages, thus ensuring a coherent and cumulative educational progression. Figure 1 summarizes the instructional method.

1. Initial Familiarization with ChatGPT
  At the beginning of the course, students were introduced to ChatGPT to develop their understanding and proficiency with the tool. This involved providing them detailed instructions on effective usage and encouraging them to engage in interactive dialogues with ChatGPT. The aim was to foster a sense of familiarity and ease, thereby facilitating an informal, hands-on learning experience.
2. Exploratory Analysis of a Dilemma using ChatGPT
  In this exploratory stage, students began to examine the topic of hospital accreditation. Through interactions with ChatGPT, they were introduced to the pros and cons of the accreditation process and to the dilemmas posed by following the accreditation guidelines. The issue of accreditation is central to the discourse on how to improve healthcare quality, but it is also fraught with challenges, such as staff shortages and funding issues. Hospitals have had to make significant changes to meet accreditation standards, leading to debates about possible abolition of the accreditation system. While accreditation is crucial for quality control, its associated costs, particularly those related to inspections and the need for additional staff, pose significant challenges. Without proportional funding, compulsory accreditation has placed financial pressures on hospitals, creating a complex dynamic for both the Ministry of Health and healthcare institutions as they navigate the accreditation process.
  To explore the topic of accreditation in depth, students were instructed to develop a series of questions to input to ChatGPT aimed at extracting detailed information about the accreditation dilemma. Students engaged with ChatGPT by posing questions and critically analyzing the answers from three perspectives: organizational, healthcare worker, and patient/customer. They iteratively refined their queries to increase precision until they achieved a comprehensive understanding. Following guidelines, they condensed and reorganized the information into a structured paragraph, incorporating the core dilemmas and arguments from each perspective. To meet objectives, students demonstrated digital media skills, including locating and sharing relevant materials, analyzing ChatGPT responses, verifying sources, and assessing content credibility.
3. Synthesis and Documentation of Concepts Emerging through ChatGPT Interaction
  In the third stage, students were required to submit a comprehensive list detailing new concepts, themes, and sub-themes that emerged from their learning experience with ChatGPT. Their submitted list was not limited to the final results, but also included documentation of all stages of their work, including their initial set of questions, their subsequent refinement of these questions, and the process of their development throughout the learning journey. In addition, they were required to provide a final section summarizing the culmination of their exploration and learning process with ChatGPT. This comprehensive approach was designed to demonstrate the students’ engagement and progression with the tool and to highlight their ability to develop their inquiries and synthesize information effectively.
4. Analytical Structuring of Learning Outcomes
  In the fourth stage, students attempted to refine the learning outcomes they had previously generated. Following the established guidelines, their main objective was to identify and highlight the pros and cons of the various arguments related to the dilemmas they had studied, making sure to consider them from different perspectives. The challenge was to present their arguments in a coherent and logical order, for example by comparing budgetary considerations with quality considerations. They were also expected to support each argument with scientific evidence, thereby aligning their analysis with academic accuracy and empirical research. This stage was crucial in developing their ability to critically evaluate and articulate complex issues, particularly in the field of healthcare.
5. Final project: Integrative Analysis and multidimensional presentation
  In the final stage, students developed and presented a final project, building upon their prior work to explore a comprehensive research question or delve into a specific aspect of their study. This included presenting organizational and managerial viewpoints. The choice of format and tools for their project and presentation—ranging from e-posters and slides to video clips, using familiar technologies like PowerPoint and ThingLink—was left to the students. This method fostered diversity and empowered students by allowing them to select their preferred presentation technique. Moreover, the project featured a peer review phase where students critiqued each other’s work through insightful questions and suggestions, enhancing the discussion. This interactive element aimed to bolster critical thinking and collaborative learning.

### Reflective Journaling: documenting the Learning Journey

Throughout the semester, students kept a reflective journal, which they submitted at the end of the course. The primary aim of this journal was to document their personal learning experiences. The journal provided a window on their challenges, difficulties and successes they encountered, all viewed through the lens of their own perceptions and experiences.

### Data analysis

The present research employed a deductive-inductive method for categorical analysis of the dataset. Integration of these deductive and inductive approaches was essential to facilitate investigation of predefined categories that are grounded in extant literature and theoretical frameworks, as well as to permit the discovery of novel categories that surfaced during the analysis process [51]. Initially, the deductive stage was conducted, focusing on predefined categories derived from existing literature and theoretical frameworks. Following this, the inductive stage allowed for the identification and development of novel categories based on the data analysis. The inclusion of episodes, thoughts, and feelings expressed by the students in this study serves to reinforce the reliability of the identified themes. The analysis of the reflective journals began with in-depth reading to identify initial themes from students’ narratives. Inductive coding facilitated the identification and development of themes by the researcher, rather than merely allowing them to ‘emerge.’ This active interpretation and organization of the data by the researcher led to a compilation of key insights. After ensuring the reliability and validity of these findings through careful review, the researcher then organized the codes into themes and sub-themes, ensuring they accurately reflected the data and provided a clear narrative of the students’ experiences.

## The findings

The researcher’s analysis of the reflective journals actively uncovered five main categories: *(1) Use of Literacy Skills; (2) User Experiences with ChatGPT; (3) ChatGPT Information Credibility; (4) Challenges and Barriers when Working with ChatGPT; (5) Mastering ChatGPT Prompting Competencies.* Table 1 summarizes the identified categories and subcategories. To further clarify each category, the table includes representative quotations from the data for illustrative purposes. Throughout the manuscript, pseudonyms have been used with quotations. This approach ensures confidentiality and anonymity for all participants.

**Table 1:** Categories, sub-categories, and representative quotations derived from the data

*Theme*	*Sub-theme*	*Example *
***Use of Literacy skills***	*Search Strategies and Access to Data in ChatGPT Use*	*The chat was super easy and helpful in making the dilemma clearer for me. It put all the info I needed in one spot, and everything was explained in a way that was simple to understand*
	*Data Analysis Enhancement with ChatGPT *	*ChatGPT really helped us out. It gave us a full picture of the whole process, including the good and bad parts, and how to handle them. We didn't even need to look at any other info sources at that point.*
	*Evaluation of Information in ChatGPT Interaction*	*ChatGPT didn't really point out which arguments were more important or less important. It kind of listed them all the same way, which made me decide for myself what to focus on. I had to pick the arguments I thought were key and then find evidence to back them up.*
***User Experiences with ChatGPT***	*Time Efficiency using ChatGPT*	*You can find out a lot about all sorts of things really quickly. The chat gives you detailed breakdowns and explanations, sorting everything into different arguments and topics; it saves you a lot of time.*
	*Accessibility and Availability of ChatGPT*	*ChatGPT is available to use anytime, anywhere using a simple and convenient interface. This would allow you to get a quick and comprehensive response at any time of the day, without having to wait around for people or experts to help you out.*
	*User-Friendly Dynamics*	*This tool is really user-friendly and easy to get. It is perfect for learning materials.*
***ChatGPT Information Credibility***		*But I have also learned that this tool has its drawbacks. It is not always right, and it certainly doesn't always give you things that are based on solid academic facts. Sometimes ChatGPT just makes things up. To be honest, realizing this was a bit of a shock to me.*
***Challenges and barriers when working with ChatGPT***	*Language Barrier*	*One big problem I had was writing in English and then translating it to Hebrew to express what I wanted to say. But I decided to take it on as a challenge and use it as a chance to improve my reading and writing in English.*
	*Technical Barriers*	*Sometimes, you can't even get into the chat because too many people are trying to use it at the same time, and other times, it just freezes up, and you can't keep using it.*
***Mastering ChatGPT- Prompting competency***		*You have to be really clear about what you are asking. It is best to give specific instructions to obtain the exact info you need. Also, you should think carefully about the answers you get, making sure the facts are right, and using your own thinking to make wise decisions.*

### Use of literacy skills

The category comprising the use of literacy skills, the code refers to instances where participants relate literacy skills such as reading comprehension, searching evaluation of Information, etc., in their interactions with ChatGPT.

It includes three subcategories: *Search Strategies and Access to Data in ChatGPT Use; Data Analysis Enhancement with ChatGPT*; and *Evaluation of Information in ChatGPT Interactions Search Strategies and Access to Data in ChatGPT Use.*

In the reflective journals, the students consistently expressed their high regard for the efficiency and ease of searching for and accessing information through ChatGPT. The chat interface significantly improved the process of retrieving information by removing the necessity to navigate through multiple websites or sources, thereby making the material more accessible. Furthermore, the interface’s user-friendly and accessible content format played a crucial role in significantly enhancing students’ understanding of the material. Shir wrote: *The chat was super easy and helpful in making the dilemma clearer for me. It put all the info I needed in one spot, and everything was explained in a way that was simple to understand.*

The analysis of the student journals underscored the remarkable proficiency of ChatGPT in rapidly and effortlessly providing information for various tasks. This technology alleviated the necessity for students to delve into multiple sources, offering a direct approach for understanding concepts, interpreting implications, and compiling data for complex issues. ChatGPT’s swift and handy information retrieval supported autonomous learning on the topic. As an accessible and user-friendly tool, it saved considerable time. Moreover, its accessibility and constant availability helped in tailoring learning experiences to fit the learner’s schedule, independent of external factors or intermediaries. ChatGPT’s use of simple, everyday language, coupled with its capacity to deconstruct and elucidate complex concepts, rendered it exceedingly approachable and beneficial for information searches and for enhancing the accessibility of educational content. Lihi also acknowledged the efficacy of ChatGPT in facilitating the rapid acquisition and expansion of her conceptual knowledge. She underscored that the ChatGPT tool obviated the need to consult multiple databases and websites for extracting conceptual information: *ChatGPT is really fast and easy to use when you need info on lots of different things. It’s great for finding technical stuff, explaining problems, understanding things better, and getting new ideas on the spot. You don’t even have to go looking for more sources – it’s all right there.*

#### Data synthesis and analysis enhancement with ChatGPT

Analysis of the reflective journals indicates that students found the synthesis, editing, and analysis of content facilitated by ChatGPT to be extremely beneficial. The tool significantly reduced the technical complexity of gathering and synthesizing information from different sources, tasks that had previously been their responsibility. As a result, they were spared the need for synthesizing, editing, and analyzing the raw data, with ChatGPT efficiently performing these functions on their behalf. Meir wrote: *ChatGPT really helped us out. It gave us a full picture of the whole process, including the good and bad parts, and how to handle them. We didn’t even need to look at any other info sources at that point*.

#### Evaluation of information in ChatGPT Interaction

The streamlined data collection procedures enabled the students to engage in more advanced learning processes, such as distinguishing between facts and assumptions, differentiating critical from non-critical information, and developing arguments as they advanced to more complex stages. The students observed that although ChatGPT presented data objectively, it did not offer explicit arguments, thus requiring them to actively interpret and formulate their own positions regarding the dilemma and identify the foundational principles for their principal arguments. For example, Miri’s reflections highlighted her need to formulate and develop a stance on the dilemma, which compelled her to engage in critical assessment of the situation:

*ChatGPT didn’t really point out which arguments were more important or less important. It kind of listed them all the same way, which made me decide for myself what to focus on. I had to pick the arguments I thought were key and then find evidence to back them up.*

Furthermore, the students were asked to support their arguments with evidence from the academic literature, necessitating a thorough evaluation and critical analysis of the information. This process led them to make informed decisions and formulate solutions. In their reflective journals, students documented a cautious approach, emphasizing the need not to simply accept information as it is presented. Instead, they highlighted the importance of thoroughly evaluating the information’s accuracy. Amir similarly addressed this issue, noting his necessity to independently navigate the “thinking part” and acquire the skills to construct strong arguments or effectively employ academic resources: *The chat didn’t really help me figure out what’s important and what’s not when I write. It also didn’t teach me how to make strong arguments or how to use academic stuff to back up my points.*

### User experiences with ChatGPT

This category refers to the qualitative data related to participants’ overall experiences, perceptions, and attitudes towards interacting with ChatGPT. The theme of user experiences is divided into three sub-themes: *Time Efficiency using ChatGPT; Accessibility and Availability of ChatGPT;* and *User-Friendly Dynamics*. Overall, analysis of the students’ reflective journals reveals broad agreement about ChatGPT’s user-friendliness and ease of use. Many students noted the chatbot’s intuitive interface and straightforward functionality, which made it accessible to those who may not be tech-savvy. This consensus highlights the effectiveness of ChatGPT as a tool that simplifies information acquisition and supports learning without the typical complexities associated with advanced technological tools.

#### Time efficiency using ChatGPT

In this sub-category, analysis of the student journals revealed the major time-saving benefits of using ChatGPT for various tasks. ChatGPT successfully eliminated the need for students to sift through numerous sources of information. By providing a straightforward way to understand a concept, grasp its implications, and gather information on complex dilemmas, ChatGPT demonstrated its efficiency in saving students’ time. Riad mentioned the significant time efficiency gained from using the tool, highlighting how it saved him considerable time: *You can find out a lot about all sorts of things really quickly. The chat gives you detailed breakdowns and explanations, sorting everything into different arguments and topics; it saves you a lot of time.*

Ali also referred to this point: *I was not very familiar with the details of accreditation, including its benefits and challenges, but within minutes I was able to grasp its essence and understand the importance of the whole process.*

The time efficiency extended not only to data retrieval and collection but also encompassed information synthesis, significantly reducing the amount of time usually required for comprehensive and coherent processing and reformulating of acquired data. Mai observed that the time saved was also because she didn’t need to search for data across multiple sources and combine it together:

*The amount of time I save is insane. If I had to search for this stuff on the internet instead of using the chat, it would take me way longer to find an answer. And even after finding it, I’d have to summarize what I found and then rephrase it in my own words, which takes so much time.*

#### Accessibility and availability of ChatGPT

A majority of the students noted that the tool’s immediate accessibility and availability significantly facilitated the personalization of learning approaches. This customization seamlessly interfaced with the unique scheduling needs of each learner, offering flexibility that in traditional learning settings is typically constrained by external factors or intermediaries. Hana highlighted ChatGPT’s anytime, anywhere accessibility through a simple interface, enabling quick and comprehensive responses without the wait for expert assistance: *ChatGPT is available to use anytime, anywhere using a simple and convenient interface. This would allow you to get a quick and comprehensive response at any time of the day, without having to wait around for people or experts to help you out.*

Lina similarly noted: *It’s pretty great how available it is (as long as it’s not too busy…). Any question I have, I get an answer. It saved me a lot of Google searches and reading articles and stuff. I get a quick and clear answer to everything I ask and it’s all super fast.*

### ChatGPT Information credibility

This category involves instances where participants discuss the credibility, reliability, and trustworthiness of the information provided by ChatGPT. Analysis of the reflective journals showed that interaction with ChatGPT facilitated students’ ability to acquire fundamental knowledge, which could then be expanded upon through subsequent inquiries and verification. Nevertheless, as students proceeded in their tasks, particularly those that required articulating arguments and substantiating their stances on complex dilemmas, they acknowledged the limitations of relying solely on ChatGPT. These limitations focused primarily on concerns about the tool’s credibility in providing sufficiently authoritative information. In this regard, Ofri appreciated ChatGPT’s quick access to information but expressed concerns over its credibility and occasional inaccuracies, leading to unexpected disappointment:

*I have found that ChatGPT has a lot of good points. It can quickly give you a lot of information on so many topics and you can really use that information. But I have also learned that this tool has its drawbacks. It is not always right, and it certainly doesn’t always give you things that are based on solid academic facts. Sometimes ChatGPT just makes things up. To be honest, realizing this was a bit of a shock to me.*

Students also noted that they were often faced with an overwhelming amount of information, some of which was irrelevant or incorrect, requiring them to evaluate the information and determine its quality. Dalia noted that while ChatGPT provided extensive information initially, aiding in learning about the topic, it also required discernment to distinguish between accurate and less relevant information: *In the first stage, the chat gave us a lot of information, which was great because it helped us learn more about the topic. But at the same time, we had to decide which information was really important and accurate and which wasn’t.*

Students’ understanding of the limitations of relying solely on the information provided to justify arguments and articulate positions in dilemmas motivated them to examine and assess its reliability. They did so by asking specific questions and consulting established academic references. From the students’ point of view, this careful research and critical evaluation process not only provided them with the opportunity to refine their powers of critical thinking and analysis, it also equipped them with the capacity to critically evaluate the credibility of the information presented. Lina wrote:

*I attempted to back up the info I found with academic sources, but then I figured out that the chat isn’t always reliable…. I went through each article that I got results from…to check where is it from, and whether the author actually existed or was just made up… After that, I did another check with other databases. This whole process made me super cautious and thorough in checking everything.*

The students expressed unanimous agreement that the need to assess the information provided by the chat forced them to be critical and use evaluation skills. Not only was this a skill they needed to be able to put to good use. It also constituted a challenge in using ChatGPT, as Limor stated that, contrary to reducing critical thinking, proper use of ChatGPT can enhance it by prompting users to reconsider and verify information, despite the challenge:

*It might seem that using ChatGPT would make you think less because, well, it’s like chatting to a robot. But actually, if you use it properly and really get into it, it adds a lot to your knowledge and makes you think more broadly and deeper. This is because it makes you think about things over and over again, and double-check the information… it wasn’t easy.*

#### Challenges and barriers in Working with ChatGPT

This category encompasses the various obstacles, difficulties, and limitations encountered by participants while using ChatGPT, including technical issues, comprehension challenges, and frustration. The analysis suggests that despite the students’ widespread agreement on the advantages of using ChatGPT, such as its ease of use, constant availability, and user-friendliness, its accompanying challenges should also be considered. Among these challenges are hesitation in adopting new, cutting-edge technology, difficulties in learning how to use the tool, and language barriers. The language issue was particularly significant, as ChatGPT operates mainly in English, which is not the first language of many of the students. Shir faced difficulties with English translation but viewed it as an opportunity to improve language skills, eventually becoming more comfortable with the chat and reducing reliance on outside translation help:

*One big problem I had was writing in English and then translating it to express what I wanted to say. But I decided to take it on as a challenge and use it as a chance to improve my reading and writing in English. Since we didn’t have to use English much, at first it felt like it took forever to understand or read stuff. But gradually, we got the hang of the chat and didn’t need as much help with translating from outside sources.*

Some students noted that they also faced some technical issues, revealing the downside of depending exclusively on online tools for studying. For many students, this was their first time using AI including applications like ChatGPT that are built on large language models. As they continued to use it, however, they became more accustomed to it. Ali found initially accessing the GPT chat difficult and, despite its ease of use, experienced issues with site access due to high traffic and occasional freezing, hindering continuous use:

*When I first tried the GPT chat for my task, it was a bit tough to get onto the site. But after a while, I noticed that even though the chat is easy to use, it’s got its problems. Sometimes, you can’t even get into the chat because too many people are trying to use it at the same time, and other times, it just freezes up, and you can’t keep using it.*

### Mastering ChatGPT-Prompting competency

This category involves instances where participants demonstrate proficiency in formulating effective prompts and questions to elicit accurate and relevant responses from ChatGPT. Analysis of the reflective journals revealed that this theme posed a notable challenge for the students, primarily due to their unfamiliarity with the tool. Indeed, they needed to learn how to use the chat effectively to elicit the correct responses and achieve their desired outcomes. Additionally, they encountered challenges in ensuring accuracy and setting the right parameters to establish a reliable and precise database. Despite these obstacles, the students recognized that their efforts to achieve accuracy and their practice of asking repetitive questions were instrumental in developing higher-order thinking skills and being able to organize and manage the required information proficiently. Liya related to this challenge by noted that dealing with inaccurate responses from the model involves clarifying questions with more details, considering alternative answers, and emphasizing the importance of verifying the information received:

*Sometimes the model may give you wrong information or answers… to cope with getting answers that are not accurate, you should make your question clearer and add more details. Also think about using different choices of answers. And it is really important to always check the answers you’re getting.*

Analysis of the reflective journals showed that systematic demonstration of these activities, along with comprehensive detailing of early learning stages and the cumulative nature of the tasks, provided students the chance to assess and revisit each step retrospectively. This reflective review allowed them to seek explanations for any aspects that were unclear, ask more questions and craft more targeted prompts, and gain a deeper understanding of the entire process. Rim, for example, explained: *The chat lets us get information in a series, like being able to ask another question to get a better understanding or clear up something from the first questions we asked. This helped us keep track of everything by linking all our questions together.*

Nir noted that the need to aim for accuracy by repeatedly refining the questions really helped in dealing with the assigned tasks effectively:

*From my experience with ChatGPT, I have learned that if you want good answers, you have to be really clear about what you are asking. You need to know what you want to achieve with the chat. It is best to give specific instructions to obtain the exact info you need. Also, you should think carefully about the answers you get, making sure the facts are right, and using your own thinking to make wise decisions.*

## Discussion

This qualitative study examined the process of introducing and using a pedagogical framework that integrates problem-based learning with the use of ChatGPT among undergraduate healthcare management students. The study also provided a qualitative exploration of their experiences using this technology and assessed how the use of ChatGPT can shift the focus from traditional digital literacy skills to advanced AI literacy skills. It demonstrated how the use of the ChatGPT platform can be managed to encourage the development of critical thinking and evaluation skills through active student engagement. These skills are considered critical for learning and working with AI platforms.

The analysis of students’ reflective journals indicated a perception of the platform as user-friendly. Minichiello et al. [54] expand the definition of “user experience” beyond mere interaction with user interfaces to include design, information presentation, technological features, and factors related to emotion, personal connection, and experience. Students described their experience with the platform positively, citing it as an incentive for ongoing engagement.

The analysis also showed that the platform’s efficiency was significantly influenced by its high availability and accessibility, which were key factors in its attractiveness to users. This attractiveness was further enhanced by its ease of use. A critical aspect of the platform’s effectiveness was its efficiency in providing key materials in a timely manner, drastically reducing the time required to retrieve information. Users particularly appreciated this aspect of the platform as it streamlined their access to information and significantly improved their learning efficiency. The platform’s ability to deliver relevant information quickly and efficiently was instrumental in its positive reception. In an academic environment where efficient time management and quick access to educational materials are essential, the platform’s ability to meet these needs effectively constituted a notable advantage.

However, students noted initial difficulties and obstacles in utilizing ChatGPT, primarily related to data credibility. These challenges, highlighted in the qualitative data, necessitated the application of critical thinking and conducting various checks to verify the information received. This concern over the credibility of information from AI tools aligns with observations by Mohamad-Hani et al. [55], who reported similar credibility issues with ChatGPT data among healthcare professionals.

Another significant challenge for the students focused on how to retrieve relevant and accurate information. To this end, they had to refine their question formulation to extract the most relevant and accurate data from the tool. Such challenges have increasingly become a focus of academic attention due to the emerging recognition of the importance of developing prompting skills for effective interaction with platforms such as ChatGPT and other AI tools [19, 20].

In terms of digital literacy skills, the findings of this study suggest that basic literacy skills such as locating, retrieving, synthesizing, and summarizing information may become less important as AI systems improve. Yet students still must be trained to evaluate and think critically about AI tools and what they can accomplish, especially since AI technologies like ChatGPT are not always completely trustworthy. Therefore, students need to learn how to evaluate the information these tools provide. These findings also offer some support for the notion that while digital literacy is undeniably recognized as crucial for the 21st century, especially in the healthcare arena [36, 45], the definition of digital literacy is changing as technological tools develop. For decades, education focused on developing basic skills. Over time, however, there was a shift toward the cultivation of more complex skills involving information evaluation, synthesis, and assessment [56, 57]. Yet as AI continues to penetrate everyday life, there has been a noticeable evolution in the forms of literacy required.

This evolution marks a transition from traditional data digital literacy, which emphasizes a basic understanding and processing of information, to AI digital literacy, which goes beyond mere data consumption to include using digital tools skillfully, understanding the nature of digital content, and effectively navigating the complex digital landscape. This shift reflects the changing demands of a technology-driven society, in which digital literacy is becoming increasingly essential for both personal and professional development [58]. As AI becomes integrated into different dimensions of work and daily life, especially in the healthcare industry, AI digital literacy will continue to evolve to meet the new demands. This will require a different set of skills, including prompting skills that allow users to better interact with AI tools [19, 20].

These results highlight the importance of rethinking the educational use of AI tools such as ChatGPT, potentially leading to changes in future learning curricula. Without the ability to use digital tools, students are liable to fall behind when it comes to adapting to new technologies, thus limiting their ability to learn key skills. Therefore, AI tools must be taught and used in a way that supports students’ holistic learning. These findings align with those of other researchers who focus on the use of the AI platform in education [40, 42, 43]. Such an approach will ensure that students are prepared for the evolving challenges and opportunities of our increasingly digital world. This is especially important in the medical education field, as AI is increasingly being used in different ways to improve the accuracy of disease diagnosis, treatment strategies, and prediction of patient outcomes [9, 10, 25, 27].

Given that AI technology is still developing and is anticipated to advance and become more widely used [21, 22], the need to adapt and acquire new literacy skills is growing. As AI evolves, reliance on traditional basic skills may decline over time, underscoring the importance of learning how to effectively utilize and interact with emerging technologies. Learning to engage with AI tools such as ChatGPT from an early stage in their education can greatly enhance students’ learning experiences. This early exposure will not only provide them with a deeper understanding of these tools. It will also boost their motivation to learn how to use them more effectively, thus highlighting the importance of training students to handle such technologies proficiently. Equally important is the need to guide students through these learning processes to ensure they acquire the necessary skills and knowledge to navigate and utilize AI tools successfully in their educational journey [11].

### Limitations and future research directions

This study utilized a pedagogical framework that integrates problem-based learning with the use of ChatGPT. While the researcher focused on the pedagogical aspect, future research is warranted to compare this digitally supported activity to a non-digital equivalent and examine the impact on students’ literacy and skills. Such a comparison would make it possible to assess what the digital instrument contributes to skill development and to identify any challenges encountered.

The use of this tool across different teaching methods could also be explored to determine whether it is particularly effective for certain types of tasks or requirements. The current study focused on health management. Implementation of this teaching approach in other academic areas should be examined to assess its effectiveness in acquiring competencies in different arenas. The findings of this study highlight the need for further research into the use of AI in learning environments that focus on goal-oriented pedagogy. Such research can help in developing educational strategies that promote the skills essential for lifelong learning.

## Conclusions and recommendations

In conclusion, revisiting the research questions in the context of our findings highlights the transformative potential of integrating ChatGPT with problem-based learning in healthcare management education. This study underscores how such integration not only shifts the focus from traditional digital literacy to advanced AI literacy skills but also enhances critical thinking and evaluation capabilities among students. These competencies are indispensable as AI continues to reshape the landscape of healthcare and medical education. AI is emerging as a transformative force that will fundamentally change the global landscape. Although we are still in the early stages of integrating and understanding AI capabilities, its potential to shape our future is clear. Adapting to this digital transformation, especially in healthcare, is crucial [4, 6].

Integrating AI into healthcare systems poses significant challenges and raises many unanswered questions [9, 10]. These issues require careful consideration and strategic planning to maximize benefits while addressing implementation complexities. The extent and impact of these transformations on the health system and its workforce remain uncertain. However, it is crucial to prepare for these changes at both individual and organizational levels. Educational institutions must update their teaching methods to meet digital demands, recognizing the critical role of educators in developing effective support strategies.

To enable healthcare professionals to integrate AI tools effectively, these tools should be introduced early in education, such as during undergraduate studies or initial professional training [9, 32, 33]. Hands-on experience allows learners to build confidence and understand the tools’ limitations. Additionally, AI tools and especially LLMs such as GPT and their applications, including platforms like ChatGPT, can serve as user-friendly and efficient learning aids, as demonstrated in this research. In addition, researchers should strive to develop innovative pedagogical methods for integrating these tools into different curricula, as exemplified here by the effective use of dilemma-based learning enhanced by ChatGPT. These studies should focus on determining which skills will become redundant and on highlighting essential competencies needed for AI literacy, including prompting, evaluation skills, and critical thinking, all of which are essential for effectively integrating AI and LLMs into medical education and daily practice. Participants in such studies have noted that the acquisition of such skills, particularly in the area of effective prompting, significantly improves the quality of AI responses. Similar to learning a new language, learning to use AI requires precise phrasing and an in-depth understanding of context. Not only will AI skills improve student engagement and comprehension, they will also encourage critical thinking, leading to better educational outcomes. Students who formulate well-structured search queries obtain more accurate responses from AI, which are critical to improving healthcare and learning outcomes.

It is therefore imperative that academia and higher education institutions, including medical education institutions, adopt methods for effectively guiding and training students in using AI. This approach is essential to address the evolving global educational landscape and to embrace the shift in roles. Educators should move from being primarily providers of knowledge to being facilitators of cultural understanding and skill development. Such a shift is essential to promote the transformative evolution of the role of educators in the modern educational context.

## Data Availability

Data are available upon request from the Corresponding author.
